# Investigation of Triple Symmetric Non-halogen Benzene Derivative Solvent for Spray-Coated Polymer Solar Cells

**DOI:** 10.3389/fchem.2021.651281

**Published:** 2021-04-23

**Authors:** Yang Tang, Hua Tang, Youjun Bai, Rong Hu, Xinwu Yan, Lu Li, Jiang Cheng

**Affiliations:** ^1^School of Materials Science and Engineering, Chongqing University of Technology, Chongqing, China; ^2^Research Institute for New Materials and Technology, Chongqing University of Arts and Sciences, Chongqing, China; ^3^Department of Engineering and Technology, Hainan College of Economics and Business, Haikou, China

**Keywords:** polymer solar cells, active layers, morphologies, ultrasonic spray coating, solvent engineering

## Abstract

The performance of spray-coated polymer solar cells could be largely improved via morphologies and phase optimization by solvent engineering. However, there is still a lack of fundamental knowledge and know-how in controlling blend morphology by using various solvents. Here, the effect of adding low polar benzene and non-halogen benzene derivatives with triple symmetric molecular has been systematically investigated and discussed. It is found that the triple symmetric non-halogen benzene could promote the formation of preferential face-on molecule orientation for PBDB-T-2Cl:IT4F films by grazing incidence wide-angle X-ray scattering. The X-ray photoelectron spectroscopy shows that PBDB-T-2Cl could be transported to the surface of the blend film during drying process. A 3D opt-digital microscope shows that triple symmetric non-halogen benzene could also improve the morphologies of active layers by reducing the coffee ring or other micro-defects. Due to the appropriate vapor pressures, devices with mixing 20% benzene or the triple symmetric non-halogen in spray solution could significantly improve the device performance. Device prepared using 20% 1,3,5-trimethylbenzene (TMB) and 80% chlorobenzene (CB) mixture solvent has the best morphology and phase structure, and the power conversion efficiency (PCE) of the device was increased nearly 60 to 10.21% compared with the device using CB as the only solvent.

## Introduction

Large-scale spray coating has gained large interest in organic electronic fabrication due to its relatively high deposition rate and accuracy in thickness control (Zhang et al., 2016, 2017, 2018c). This technique has been widely used for laboratory polymer solar cell fabrication, especially some special situations such as flexible, textile, semi-transparent devices, and large-scale modules (La Notte et al., 2018; Zhai et al., 2018; Li et al., 2019). In the past years, polymer solar cells fabricated by spray coating have induced a qualitative leap, which is attributed to the up-gradation of material and improved processing. High-efficiency devices in lab-scale have been prepared by many groups, e.g., Zhang et al. obtained conventional and inverted PffBT4T-2OD:PC_71_BM devices with power conversion efficiency (PCE) of 8.13 and 8.43%, respectively (Zhang et al., 2017). Although this result is far from lab-scale devices prepared by spin-coating, the prospect in industrial large-scale fabrication for spray coating is more competitive. For example, it has been reported that a very high PCE of 8.06% was achieved by a high-efficiency large-scale module (25 cm^2^) consisting of spray cast active layer and PEDOT: PSS (Zhang et al., 2015).

For the processing of a high-efficiency large-scale device, a good topography with fewer micro-defects such as stripe, coffee rings, and pinholes is very essential (Krebs et al., 2011; Lee et al., 2013; Lei et al., 2019). Additionally, the comfortable phase structure including suitable domain size and vertical bi-continuous interpenetrating networks in the active layer could largely improve transport efficiency of the charge carrier and photovoltaic performance (Dkhil et al., 2017; Yao et al., 2018). To achieve these goals, chemical additives like 1,8-diiodooctane (DIO), diphenyl ether (DPE), and 1-chloronaphthalene (CN) are widely used to optimize the morphology in many coating methods, although it could bring some uncertainty impact in thermal stability or lifetime (Park et al., 2018; Zhang et al., 2018b; Yuan et al., 2019). For spray deposition, the film quality also depends on the physical state (i.e., solubility, viscosity, and vapor pressure) of the spray solution. In our previous work, the PCE of spray-coated PTB7:PC_71_BM solar cells had been improved from 6.11 to 8.23% by tuning the drying processes (Cheng et al., 2018). We have also demonstrated the PBDB-T-2Cl: IT4F device with a very high efficiency of 12.29%, fabricated via piecewise controlling spray, that is with donor/acceptor ratio 1:2 and 2:1 in the coating sequence (Cheng et al., 2020). In the active layer deposition process, the solvent, TMB, plays a primary role in solvent engineering. By adding this solvent, the active layer tends to format a vertical phase-separated structure accompanied by a significant reduction in defects related to the Marangoni effect such as coffee rings and pinholes. Although the progress is notable, the fundamental knowledge for this solvent is not clearly understood yet, and the know-how in controlling the morphology for active layers remains unknown to the research community.

As we know, the TMB molecule is triple symmetric, exhibiting very low polarity. It has a higher boiling point and also lower toxicity than halogen benzene. To further investigate the fundamental mechanism of effect, this solvent has, on active layers as well as device performance, the effect of different triple symmetric non-halogen benzene derivative solvents, including benzene, and TMB, and 1,3,5-triethylbenzeneand (TEB) has been studied as well. As a result, we found that triple symmetric non-halogen benzene has various effects on the morphologies, domain size, molecule orientation, and vertical component distribution of active layers. All the devices show obvious improvement by using the solvent. The devices using 20% TMB and 80% CB mixture solvent show the best performance, resulting in a high PCE up to 10.21% for ultrasonic spray-coated PBDB-T-2Cl: IT4F solar cells.

## Results and Discussion

**Figure 1:**
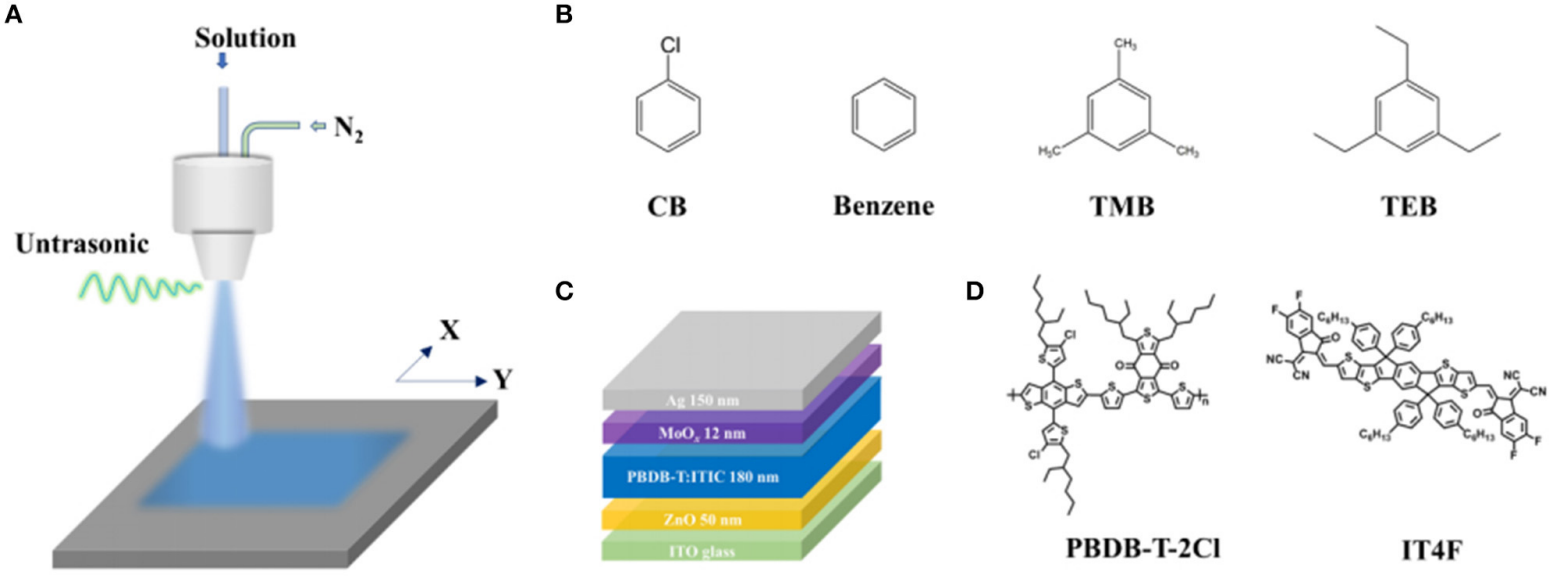
Spray preparation of PBDB-T-2Cl:IT4F photovoltaic device. **(A)** Schematic of ultrasonic spray apparatus, **(B)** molecular structures of chlorobenzene (CB), benzene, 1,3,5-trimethylbenzene (TMB), and 1,3,5-triethylbenzene (TEB), **(C)** device structure, and **(D)** molecular structures of PBDB-T-2Cl (donor) and IT4F.

The schematic of the spray apparatus consisted of an ultrasonic nozzle, XY table, and pipe fitting is depicted in Figure 1A. The molecule structures of CB, benzene, TMB, and TEB are shown in Figure 1B, while the device structures, PBDB-T-2Cl (donor) and IT4F (acceptor), are presented in Figures 1C,D, respectively (Cheng et al., 2016, 2018). All the samples were prepared in the same condition except the solvent composition. To fully dissolve both donor and acceptor, the spray solution contained 80 vol% CB and 20 vol% triple symmetric non-halogen benzene. To simplify the discussion, there are no other additives in the spray solution. To avoid enormous irregularity, four specimens were fabricated for each technique. Each specimen has eight devices with a 4-mm^2^ active area. All specimens were characterized and measured under the same circumstances and procedures.

**Figure 2:**
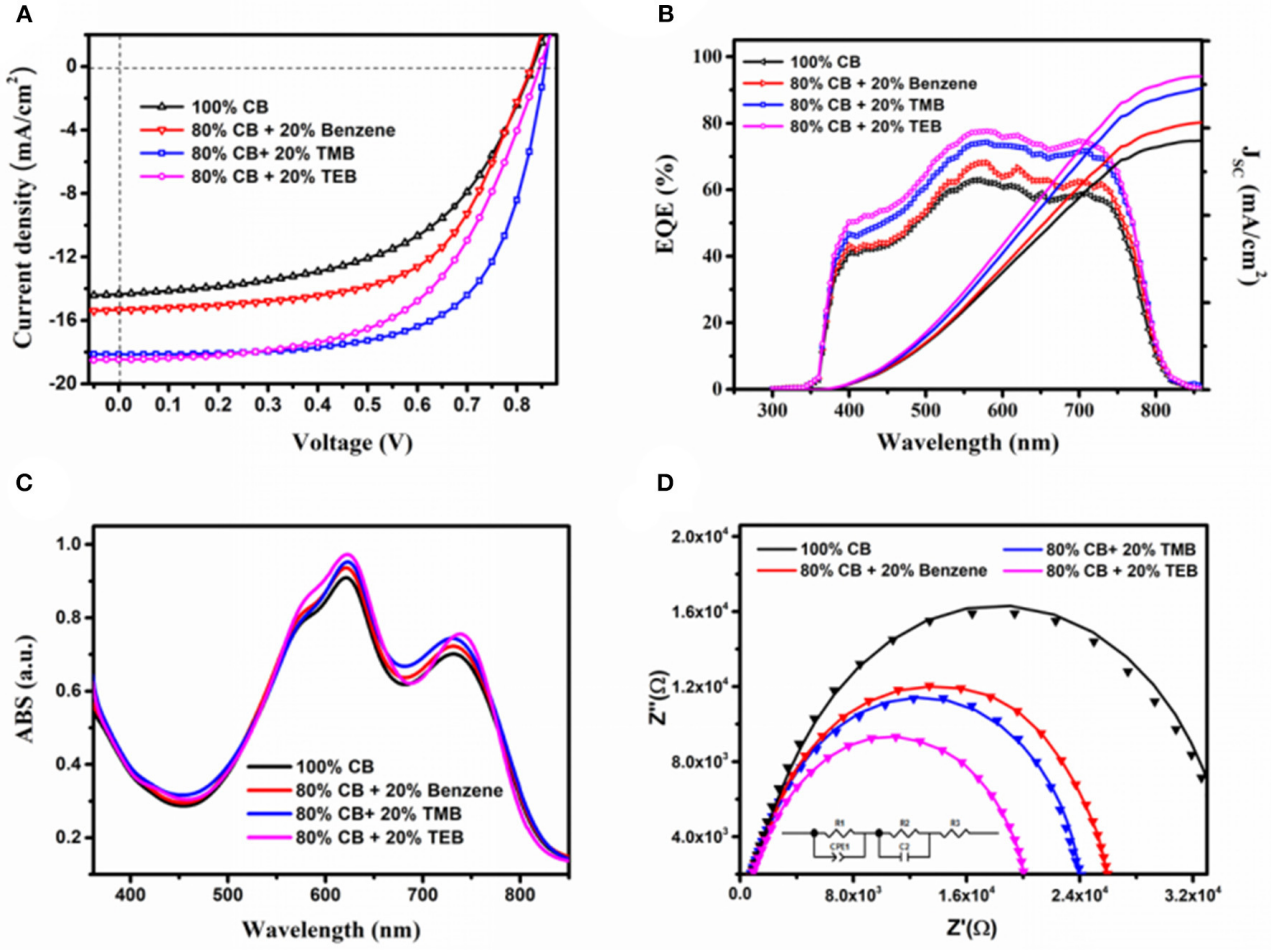
Performance of spray-coated devices by varied solvents. **(A)** J–V characteristics, **(B)** EQE curve, **(C)** absorption spectra, and **(D)** Cole–Cole plots, solid line represents the fitted line.

The J–V characteristics and external quantum efficiency known as EQE curves of the devices prepared by ultrasonic spray using mixture solvent containing 80 vol% CB and 20% triple symmetric non-halogen benzene are illustrated in Figures 2A,B, and devices performances are presented in Table 1. The control device prepared by ultrasonic spray using 100% CB exhibits a J_SC_ of 14.35 mA/cm^2^, a V_OC_ of 0.83 V, and an FF of 53.70%, leading to a relatively low PCE of 6.41%. Overall, all the devices using benzene and triple symmetric non-halogen benzene show better device performance compared with the control devices. The devices prepared using a mixture solvent containing 80% CB and 20% benzene exhibit significant improvements in performance, that is, a J_SC_ of 15.34 mA/cm^2^, an FF of 59.51%, thus resulting in an improved PCE of 7.59%. When 80% CB and 20% TMB was used, the devices show the best performance. The PCE was increased to 10.21% resulting from a high J_SC_ of 18.14 mA/cm^2^ and an FF of 65.68%. The devices using 80% CB and 20% TEB also show high performance because of the highest J_SC_ of 18.43 mA/cm^2^. Nevertheless, the value of PCE is only about 8.86% that is a little lower than that using TMB solvent because of its lower FF of 56.74%.

**Table 1:** Performance of devices prepared by ultrasonic spray using varied solvents.

**Sample**	**Solvent**	**V_**OC**_ (V)**	**J_**SC**_ (mA/cm^**2**^)**	**FF (%)**	**RS (Ω cm^**2**^)**	**PCE (%)**
Device 1	100% CB	0.83 ± 0.01	14.35 ± 0.42	53.70 ± 2.12	15.48 ± 1.65	6.41 ± 0.17
Device 2	20% benzene	0.83 ± 0.02	15.34 ± 0.49	59.51 ± 1.73	15.62 ± 2.17	7.59 ± 0.23
Device 3	20% TMB	0.85 ± 0.02	18.14 ± 0.53	65.68 ± 1.78	6.65 ± 3.23	10.21 ± 0.29
Device 4	20% TEB	0.85 ± 0.01	18.43 ± 0.68	56.74 ± 1.48	10.58 ± 2.02	8.86 ± 0.31

*PCE, power conversion efficiency*.

The EQE spectrum shown in Figure 2B demonstrates an analogous trend with the J_SC_ of the devices using varied solvents. The shape of EQE curves resembles the previous reported PBDB-T-2Cl:IT4F solar cells. The J_SC_ values of the devices using 100% CB, 20% benzene, 20% TMB, and 20% TEB calculated from EQE spectrum are 14.42, 15.41, 17.39, and 18.09 mA/cm^2^, respectively, which are agreeing with the J-V characteristics. The enhanced EQE indicates that the devices using benzene and triple symmetric non-halogen benzene had different charge carrier dynamics, which include carrier transmission and the recombination with control devices. Figure 2C shows the absorption spectra of the blend films prepared by varied solvents. As we see, the difference of absorption was very small that could not bring much effect on the performance of the devices. Obviously, the enhancement of device performance mainly comes from the improving of the morphology and interface when using the triple symmetric benzene, and bringing a visible increase in J_SC_ and FF. The value of V_OC_ also has some slight improvement because of the less recombination. However, the V_OC_ here is mainly contributed by the energy level of LUMO and HOMO, and the increase is small.

Frequency-dependent impedance has been employed to analyze the carrier dynamics effected by triple symmetric non-halogen benzene, and we modeled it by an equivalent circuit. The Cole–Cole plots, fitted results, and element equivalent circuit are illustrated in Figure 2C. All parameters calculated from the fitted curves are tabulated in Table 2. The Cole–Cole plot, which represents the carrier recombination process of each device, is a quasi-semicircle. The real impedances Z' has identical tendency with J_SC_ as the solvent varies, implying that the conductivity of the devices with benzene and triple symmetric non-halogen benzene are higher than the control device. In the equivalent circuit model, the resistor R1 and a constant phase element (CPE1) are used to represent factors that influence the carrier transmission from the PBDB-T-2Cl/IT4F interface to the electrode. The CPE could seem as a non-ideal capacitance defined by two values: CPE-T (the capacitance) and CPE-P (a non-ideal capacitor) (Arredondo et al., 2014; Zheng et al., 2016). The shunt pair R2 and C2 are defined as two electrical contacts (a resistance with a parallel capacitance) of the interfaces between the active layer and buffer layer, and the group R2C2 represents all the factors that affect charge collection on the interface. R3 is all the electrode resistances including buffer layers, ITO, and Ag. The differences of the elements R2, C2, and R3 for different samples are not significant, indicating that the effect of the solvent on carrier transfer in the active layer is dominant. As shown in Table 2, R1 for devices using benzene, TMB, and TEB shows an obvious decrease, while the CPE1-T for them is much higher than the control device. The voltage-dependent time constant (τ_avg_) represents the carrier recombination lifetime and is dependent on the nature of the interfaces distributed between the acceptor and the donor. It can be approximately calculated by Equation 1.

(1) $$\begin{array}{r} \tau_{\text{avg}}\;=\text{ CPE}1-\text{T }\times \;R1 \end{array}$$

**Table 2:** Calculation result of equivalent circuit parameters from the fitted impedance spectrum.

**Sample**	**Solvent**	**R1 (Ω)**	**CPE1-T (10^**−9**^ F)**	**CPE1-P**	**R2 (Ω)**	**C2 (10^**−9**^F)**	**R3 (Ω)**	**τ_avg_ (μs)**
Device 1	100% CB	23,976	4.05	0.96	694	5.25	254	97
Device 2	20% benzene	23,137	4.14	0.96	669	5.42	224	96
Device 3	20% TMB	22,648	4.86	0.95	596	5.47	245	110
Device 4	20% TEB	23,289	4.39	0.98	850	4.96	213	102

*CB, chlorobenzene; TMB, 1,3,5-trimethylbenzene; TEB, 1,3,5-triethylbenzene; CPE1, constant phase element*.

**Figure 3:**
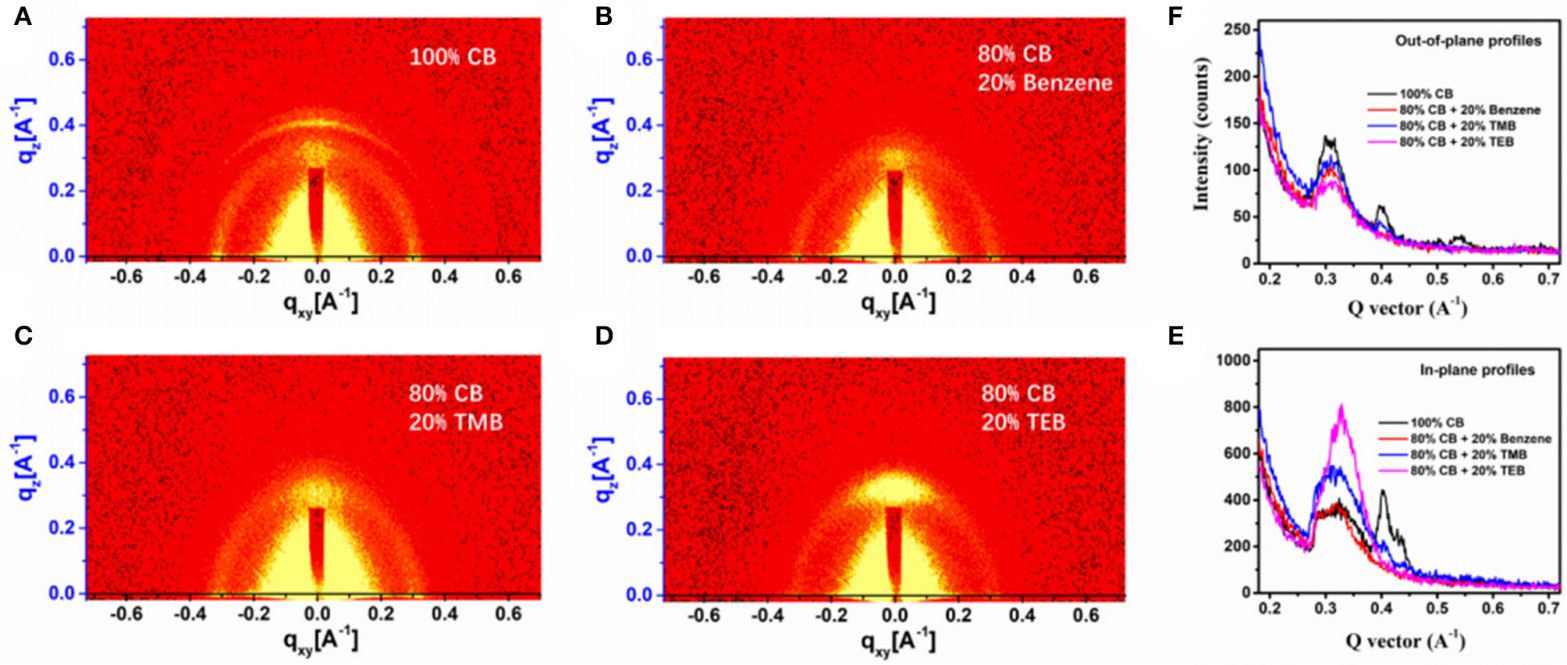
Grazing incidence wide-angle X-ray scattering (GIWAXS) images of the films prepared using **(A)** 100% CB, **(B)** 80% CB + 20% benzene, **(C)** 80% CB + 20% TMB, and **(D)** 80% CB + 0% TEB; **(E)** out-of-plane and **(F)** in-plane GIWAXS profiles for devices using varied solvents.

The device that is using 20% TMB has the highest τ_avg_ of 110 μs, indicating that the sample has the poorest carrier recombination (Yao et al., 2014; Lin et al., 2017). The grazing incidence wide-angle X-ray scattering (GIWAXS) was used to investigate the phase structure of PBDB-T-2Cl:IT4F blend made from triple symmetric non-halogen benzene. The 2D GIWAXS pictures of the PBDB-T-2Cl:IT4F blend films, which was fabricated with 100% CB, 20% benzene, 20% TMB, and 20% TEB, respectively, are demonstrated in Figures 3A–D. Figure 3E shows the intensity profile of the out-of-plane orientations, in which the diffraction peaks represent the number of edge-on molecules, while Figure 3F corresponds to in-plane orientations, and the diffraction peaks represent the number of face-on molecules (Yang et al., 2019; Ma et al., 2020). As shown in the profiles, the film prepared using 100% CB shows several peaks at around 0.31, 0.40, and 0.54 Å^−1^ at out-of-plane, and two peaks at 0.34 and 0.42 Å^−1^ at in-plane, exhibiting the features of molecule disorientation. When using 20% benzene, the orientation is improved. It shows only two obvious peaks at around 0.31 Å^−1^ at out-of-plane and 0.34 Å^−1^ at in-plane. As seen in Figure 3F, the peaks at 0.34 Å^−1^ for blend films prepared using TMB and TEB is much higher than the other, exhibiting their preferential face-on orientation. The film prepared using TEB has the best face-on orientation obviously. The increasing J_SC_ and PCE values of the device prepared using benzene and triple symmetric non-halogen benzene are also probably attributed to the increasing orientation of the molecules since the face-on orientation is beneficial to the transportation of free carriers toward contact electrodes (Luo et al., 2018; Qiuju et al., 2018).

**Figure 4:**
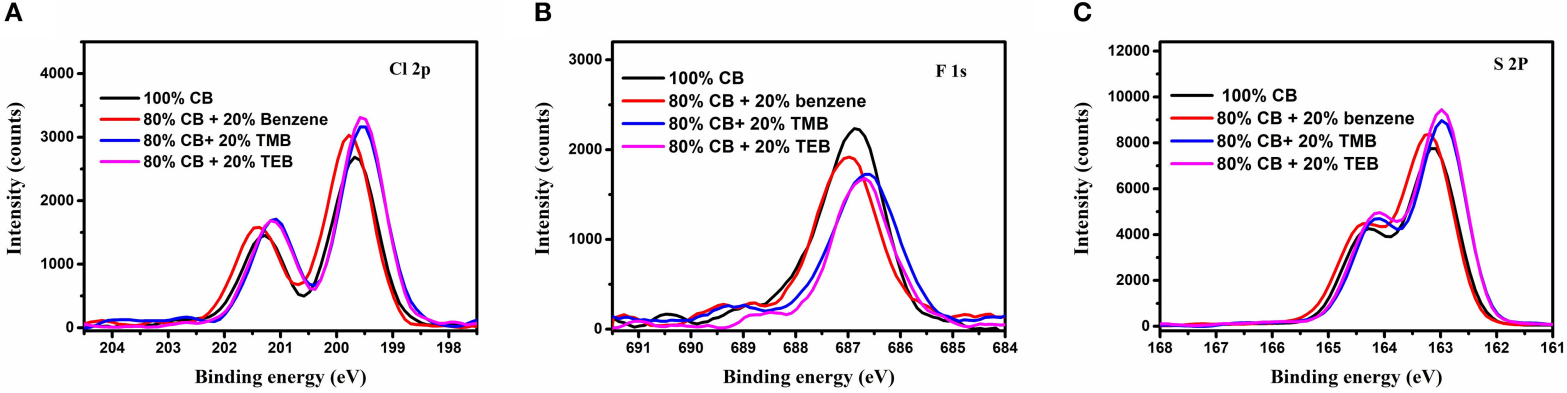
**(A)** C1s, **(B)** F1s, and **(C)** S2p X-ray photoelectron spectroscopy (XPS) peaks of the films using varied solvents.

It is well-known that the formation of molecule orientation for polymer films is usually a self-assembly process. There should be some solute transport during the growth of blend films. In order to study the mass transport, elemental distribution in PBDB-T-2Cl:IT4F blend films prepared using different solvents were examined by X-ray photoelectron spectroscopy (XPS) (Zhao et al., 2017; Borges et al., 2018). To avoid the influence of surface contamination, all the samples were gently etched by plasma, and the XPS spectra of Cl2p, F1s, and S2s are depicted in Figures 4A–C, respectively. The Cl2p peaks for films using benzene, TMB, and TEB are much higher than those using 100% CB, while the intensity of F1s is lower. Due to the fact that Cl only exists in donor and F only exists in acceptor, this result indicates that more PBDB-T-2Cl have been transported to the surface of blend film when using benzene and triple symmetric non-halogen benzene. Among the mentioned solvents above, the transport rate for the sample using TEB is the highest. Besides, the variation trend of S2p consists of Cl2p as well, probably due to PBDB-T-2Cl having a higher S ratio (22.2%) than IT-4F (8.96%), which further confirms our point. Making careful observations, XPS core levels of films prepared using triple symmetric benzene, TMB, and TEB show a clear shift compared with the film prepared using 100% CB, which could also provide some chemical information. Interestingly, the shift tendencies of Cl2p, F1s, and S2p XPS peaks were all the same. Take Cl2p peaks as an example: the binding energy of Cl2p_3/2_ XPS peaks of the film prepared using 100% CB is centered at 199.65 eV, and it shows a tiny shift to 199.78 eV when using 80% CB and 20% benzene mixture. The binding energies of the films using 20% TMB and TEB show an obvious shift to lower energy, and both of them are peaked at 199.53 eV. Although there is a lack of direct evidence, we believe the cause of XPS shift was related to the variation of domain component or molecule stacking state, which could affect the outer electron cloud density because there was no other component that could be introduced using different solvents.

C1s(A),F1s(B), and S2p(C) X ray photoelectron spectroscopy(XPS) peaks of the films using varied solvents.

**Figure 5:**
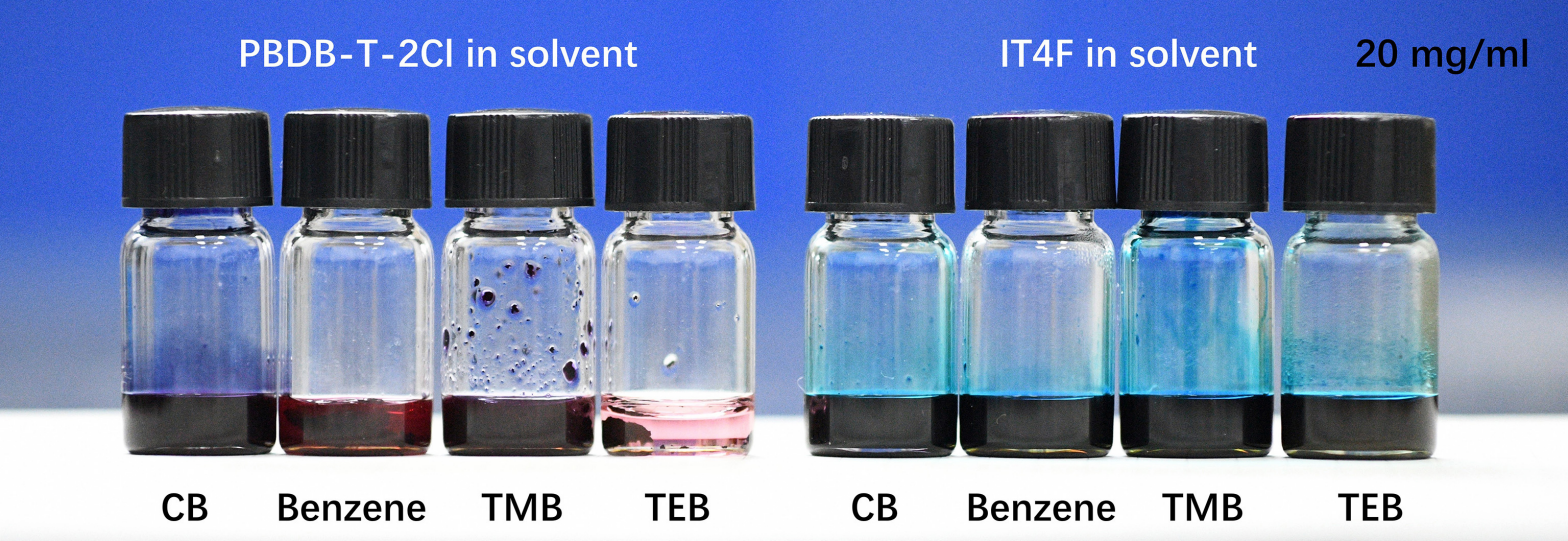
The solubility of IT4F and PBDB-T-2Cl in CB, benzene, TMB, and TEB. IT4F could easily be dissolved in benzene, TMB, and TEB, while PBDB-T-2Cl has a very low solubility in benzene, TMB, and TEB.

The traces of the driving force for self-assembled and solute transport could be found in the drying process effected by the solvent component. Due to the principle of the dissolution in a similar material structure, the non-polar benzene, TMB, and TEB are good solvents for IT4F but bad solvents for PBDB-T-2Cl at normal temperature as shown in Figure 5. Solubility test results show that the solubility of IT4F in benzene, TMB, and TEB are approximately higher than 60 mg/ml, while the solubility of PBDB-T-2Cl in them are lower than 10 mg/ml. The solvent used in our experiment shows significant differences in volatility in air at room temperature. Vapor pressures of CB, benzene, and TMB are 11.8, 74.6, and 2.5 mmHg, respectively, at 25°C, and TEB has a very low volatility at room temperature, the vapor pressure of which is even lower than measurement limits.

**Figure 6:**
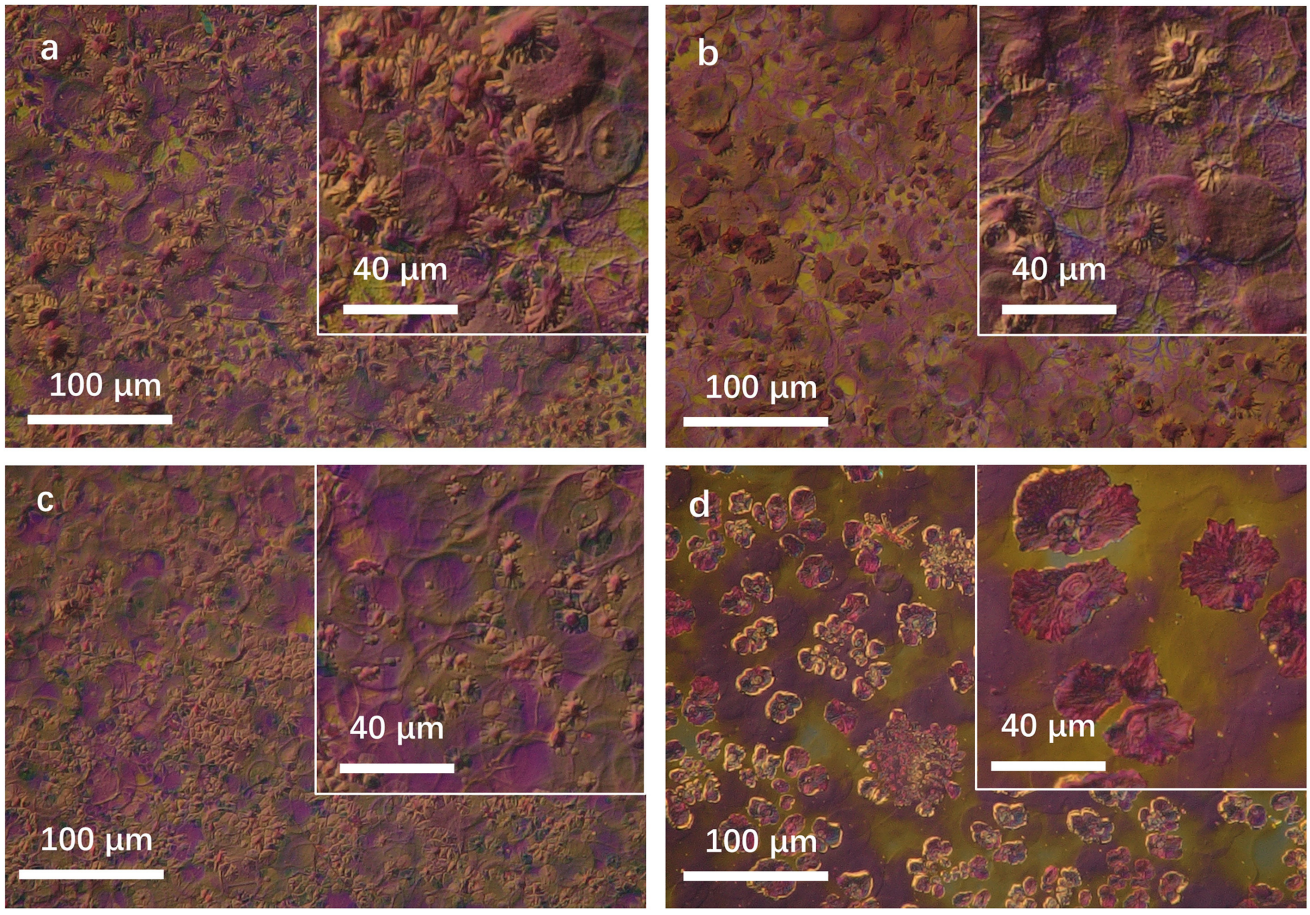
3D opt-digital microscope image for PBDB-T-2Cl:IT4F blend films using varied solvents. **(a)** 100% CB, **(b)** 80% CB + 20% benzene, **(c)** 80% CB + 20% TMB, and **(d)** 80% CB + 0% TEB.

As we know, benzene has a much higher vapor pressure than CB. It could be volatilized rapidly during the growth of the film. The effect on morphology is merely the improvement of solubility for IT4F and the crystallization rate of domains. Thus, the performance of the device prepared using CB shows a limited improvement, as shown in Figure 2; Table 1. In comparison, TMB and TEB have a much lower volatility rate than CB at room temperate. When the spray solution consists of 80% CB and 20% TMB or TEB, CB would be first volatilized, and then PBDB-T-2Cl-rich domains would precipitate at the surface and form a network structure extended from the surface to the body, and IT4F was located in a deeper position, that is, the blend film would form a clear vertical phase separation structure, providing an efficient potential pathway for positive/negative carriers to transport and be collected by electrodes (Li et al., 2013; Wu et al., 2015). Accordingly, the performance of the device prepared using TMB or TEB shows a considerable improvement. The drying process also has a significant effect on the surface morphology of blend films. Figure 6 has illustrated the 3-D digital optical microscope of images for spray coated PBDB-T-2Cl:IT4F blend films prepared by using varied solvents. The blend films using 100% CB and 80% CB and 20% benzene showed a rapid drying process, leading to a heavy “coffee-ring” effect and rough surface morphology as shown in Figures 6a,b, the mechanism of which has been detailed discussed in our previous report (Cheng et al., 2016). Nevertheless, both the density and the height edge of coffee-rings reduced tremendously when using 20% TMB or TEB. Particularly, the coffee-ring in the sample using 80% CB and 20% TMB even were hardly observed. Unfortunately, many IT4F-riched aggregates could be found in some regions of blend film due to the high solubility of TEB for IT4F as well as its low vapor pressure at room temperature in air. The aggregates should bring some interfacial problems that affect much to the carrier transport and could bring obvious degradation for device performance.

**Figure 7:**
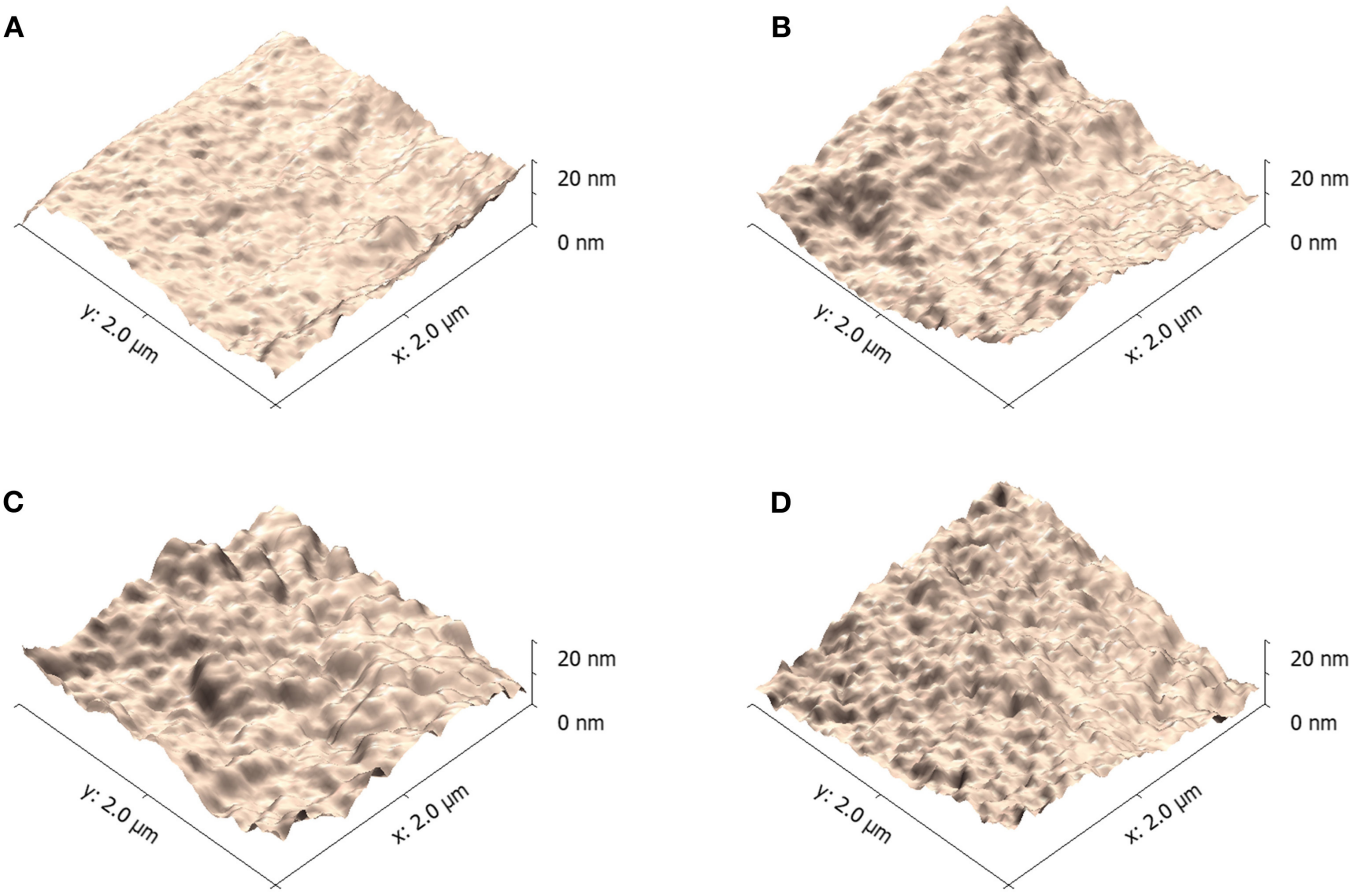
Atomic force microscope (AFM) (2 μm × 2 μm scale) imaging for PBDB-T-2Cl:IT4F blend films using varied solvents. **(A)** 100% CB, **(B)** 80% CB + 20% benzene, **(C)** 80% CB + 20% TMB, and **(D)** 80% CB + 0% TEB.

The triple symmetric non-halogen benzene derivatives also have some effect on the domain of PBDB-T-2Cl:IT4F blend films. AFM was applied to examine the nano-scale morphology of the blend films influenced by varied solvents (Zhang et al., 2018a; Fan et al., 2019). As shown in Figure 7, blend film prepared using 100% CB has small aggregates with low root-mean-square roughness (RMS) value of 5.33 nm. The RMS values of blend films prepared using benzene, TMB, and TEB are 6.28, 10.62, and 9.40 nm, respectively. We noticed that the blend films prepared using TMB and TEB show significant aggregates. As we previously reported, an appropriately large size of aggregates is helpful to improve the device performance (Cheng et al., 2018). The larger aggregates reflect a higher crystallinity, indicating that the blend films have experienced a long crystallization period, which is beneficial for the formatting of phase separation structure and constructing of an interpenetrating network (Liang et al., 2018). This structure could provide smooth pathways for carriers to transport to electrodes in bulk heterojunction solar cells. The formation of aggregate is always affected by solvent volatilization rate and solute transfer rate. By adding 20% TMB to CB, it could effectively reduce the fast volatilization of CB, proving a long-enough time for crystallization. However, if the viscosity of additive was too large, the crystallization could also be restricted. Here, the blend films prepared using TMB has the largest aggregates distributed across the surface, corresponding to the best phase separation and crystallinity, resulting in the best performance for devices.

## Experimental Section

Non-fullerene acceptor IT4F and conjugated polymer PBDB-T-2Cl were bought from Solarmer Material Inc (Beijing). The cathode buffer layer (CBL) is made up of ZnO (50 nm) by spray pyrolysis. Patterned ITO glass was used as the substrate with a sheet resistance of 10 Ω/sq. Active layers, 180-nm thick, were then spray-coated with PBDB-T-2Cl:IT4F solution (1:1 of D/A ratio, 3 mg ml^−1^ of the polymer concentration) in air. During coating, the solution-spraying rate and the flow rate of the carrier gas (N_2_) were kept at 0.085 ml/m and 20 L/min, respectively. The devices were finished by thermal evaporating MoO_3_ (12 nm) as an anode buffer layer (ABL) and Ag (150 nm) as an anode under vacuum below 10^−4^ Pa with shadow masks above. Each one of the specimens had four cells with 4-mm^2^ active areas.

The current density voltage, also known as J–V characteristics of the devices, was investigated under a xenon lamp illumination (94043A, Newport), the power density of which is 100 mW/cm^2^, and a Keithley 2400 source meter was used to record the data. An electrochemical workstation (Chen Hua CHI660E, China) was used to measure frequency-dependent impedance in the frequency range of 1 Hz to 1 MHz in dark space. An integrated system (Beijing SOFN 7-SCSpecIII) with a lock-in amplifier was used to measure external quantum efficiency (EQE) under short-circuit conditions.

The surface morphology of the PBDB-T-2Cl:IT4F films was observed under an atomic force microscope (AFM, Agilent 5500) and a 3-D digital optical microscope (Olympus DSX500, Japan). A stylus profile meter (Alpha-Step D-100, USA) was used to measure the thickness of the films. The X-ray photoelectron spectroscopy (XPS, Thermo ESCALAB, USA) was used to characterize the chemical composition distribution of the films. The molecular orientation of blend films was investigated by GIWAXS using a Bruker Nanostar system (AXS GmbH, Germany).

## Conclusion

To summarize, we have investigated the effect of the triple symmetric non-halogen benzene, TMB, and TEB on the performance of PBDB-T-2Cl:IT4F solar cells. Due to the significant difference of solubility to donor and acceptor material, and their different vapor pressures at room temperature, the mixture with a different solvent has many effects on the morphologies, domain size, molecule orientation, and component distribution of active layers. All the devices with mixing 20% benzene or the triple symmetric non-halogen in spray solution could significantly improve the device performance. Particularly, TMB and TEB could largely improve the device performance (34–59% PCE increasing). Overall, due to TMB that has a more appropriate vapor pressure at room temperature, the device prepared using 20% TMB and 80% CB mixture solvent has a comfortable morphology with less coffee ring or other micro-defects. Using this mixture solvent, the PCE for spray-prepared devices has exceeded 10%, showing a good prospect in large-scale fabrication of polymer solar cells.

## Data Availability Statement

The original contributions presented in the study are included in the article/supplementary material, further inquiries can be directed to the corresponding author/s.

## Author Contributions

JC and LL contributed to the conception of the study. YT performed the experiment. RH and XY contributed significantly to analysis and manuscript preparation. HT and JC performed the data analyses and wrote the manuscript. YB helped perform the analysis with constructive discussions. All authors contributed to the article and approved the submitted version.

## Conflict of Interest

The authors declare that the research was conducted in the absence of any commercial or financial relationships that could be construed as a potential conflict of interest.
